# A Randomized, Double‐Blinded, Placebo‐Controlled QTc Study to Evaluate BIA 28–6156 Effect on Cardiac Repolarization in Healthy Volunteers

**DOI:** 10.1002/cpdd.70055

**Published:** 2026-04-09

**Authors:** Isa Peixoto, Dana Hilt, Teresa Carvalho, João Reis, Helena Gama, Joerg Holenz

**Affiliations:** ^1^ Research & Development BIAL‐Portela & C^a^, S.A Porto Portugal; ^2^ Actinogen Medical Sydney Australia

**Keywords:** BIA 28–6156, cardiac repolarization, QT, QTc, GBA activator

## Abstract

Mutations in the *GBA1* gene, encoding beta‐glucocerebrosidase (GCase), are the most common genetic risk factor for Parkinson's Disease (PD). BIA 28–6156, an allosteric activator of GCase, is under development for the treatment of GBA‐associated PD. This Phase 1, randomized, double‐blind, placebo‐controlled, crossover study assessed the impact of BIA 28–6156 on QT interval corrected (QTc) for heart rate (HR) based on the Fridericia correction (QTcF) in 37 healthy subjects. Participants received single doses of 60 or 150 mg BIA 28–6156, 400 mg moxifloxacin, or placebo in a cross‐over design. The relationship between BIA 28–6156 plasma levels and QTcF changes (ΔQTcF) was analyzed to exclude a ΔΔQTcF > 10 ms. Heart rate, PR, and QRS intervals, electrocardiogram (ECG) waveform morphology, and adverse events (AEs) were also evaluated. The concentration‐QTc analysis indicated no effect on ΔΔQTcF exceeding 10 ms up to BIA 28–6156 plasma levels of ≈7150 ng/mL. No clinically significant effects on ECG parameters were observed, and BIA 28–6156 was generally well tolerated, with no deaths or serious AEs. This study concluded that BIA 28–6156 has no clinically relevant impact on ECG parameters, confirming a negative thorough QT/QTc study.

Beta‐glucocerebrosidase (GCase) is a lysosomal enzyme, encoded by the *GBA1* gene, responsible for hydrolyzing the glycosphingolipid glucosylceramide into glucose and ceramide.^1^ Additionally, GCase can hydrolyze glucosylsphingosine into glucose and sphingosine.^2^, ^3^

*GBA1* mutations lead to decreased GCase activity, associated with endoplasmic reticulum stress, inflammation, α‐synuclein aggregation in dopaminergic neurons, and ultimately cell death.^4^

Homozygous mutations in the *GBA1* gene that result in significantly reduced GCase activity are responsible for causing Gaucher disease (GD),^5^, ^6^ a lysosomal storage disorder characterized by impaired glycosphingolipid degradation. This impairment leads to abnormal substrate accumulation, disrupting cellular signaling pathways, calcium homeostasis, and intracellular trafficking, with manifestations in various tissues, including viscera, bone, bone marrow, and, in some cases, the brain.^5^

*GBA1* variants can be classified according to their biochemical effect on GCase enzymatic activity. Mild variants have an in vitro residual enzymatic activity of 32%–38% and are associated with Gaucher disease (GD) type I in homozygous/compound heterozygous carriers, whilst severe variants have an in vitro residual activity of 13%–24% and are associated with GD type II and III in homozygous/compound heterozygous carriers.^7^

*GBA1* mutations not only may lead to GD but are also the most common genetic risk factor for Parkinsonism in several clinical settings. The presence of one abnormal GCase allele (heterozygous mutation) is a risk factor for GBA‐PD.^8^

At presentation, symptoms in individuals with *GBA1* mutations are clinically indistinguishable from idiopathic Parkinsonism (iPD), typically involving at least two of the three cardinal features: bradykinesia, rigidity, and/or tremor. While there is no difference between the two groups of patients regarding tremor, GBA‐PD patients were shown to exhibit faster cognitive and motor deterioration than iPD patients.^9^

Compared to iPD, GBA‐PD usually presents an earlier age of onset, and disease progression in GBA‐PD is usually more rapid,^10^, ^11^ with a shorter survival from the time of diagnosis,^12^ higher prevalence of cognitive dysfunction,^13^, ^14^ and a higher occurrence of dementia.^10^, ^11^, ^15^

GBA‐PD is further characterized by a greater prevalence of depression, anxiety, hallucinations, and rapid eye movement sleep behavior disorder.^16^, ^17^ Notably, *GBA1* mutations are the only known Parkinson's disease risk factor that accelerates disease progression.^18^

Current treatments for iPD focus on symptomatic relief by enhancing dopaminergic function, typically through pharmacological interventions like levodopa or dopamine receptor agonists, or deep brain stimulation. However, these treatments do not modify disease progression and may be contraindicated in GBA‐PD due to the potential for exacerbating cognitive decline.^19^, ^20^

Therefore, there is a significant unmet medical need for therapies that target the underlying pathogenesis of GBA‐PD and would be able to modify the course of the disease, whilst not only providing symptomatic benefit.

BIA 28–6156 is an allosteric activator of GCase under clinical development as a potential treatment for GBA‐PD.^21^, ^22^ The enhancement of the GCase activity, through allosteric modulation, could provide disease modification effects in patients with a heterozygous *GBA1* mutation, which was already supported by available pre‐clinical data (data on file). As such, BIA 28–6156 is a possible novel, first‐in‐class potential treatment for patients with GBA‐PD.

QT prolongation is an established marker of a drug's proarrhythmic risk.^23^, ^24^ QT interval prolongation can lead to fatal arrhythmias,^25^ with QTc increases higher than 20 ms posing a definite torsade de pointes risk.^26^

The objective of this study was to evaluate the effect of single therapeutic (60 mg) and single supratherapeutic (150 mg) oral doses of BIA 28–6156 on QT interval with Fridericia correction (QTcF) for heart rate (HR) in healthy subjects.

## Methods

### Study Design

This was a Phase 1, randomized, double‐blinded, placebo‐controlled, single‐dose, 4‐period crossover study (trial registration EudraCT number: 2021‐002546‐32) to evaluate the effect of BIA 28–6156 on cardiac repolarization in healthy male and female subjects. A total of 37 healthy subjects were randomized into 1 of 12 treatment sequences using a 3‐Williams squares design (Table 1).

**Table 1:** Overview of Study Treatment Periods and Treatment Sequences

Treatment sequence (n)	Treatment period 1	Treatment period 2	Treatment period 3	Treatment period 4
1 (n = 3)	B	C	A	D
2 (n = 3)	C	D	B	A
3 (n = 3)	A	B	D	C
4 (n = 3)	D	A	C	B
5 (n = 4)*	C	A	B	D
6 (n = 3)	A	D	C	B
7 (n = 3)	B	C	D	A
8 (n = 3)	D	B	A	C
9 (n = 3)	A	B	C	D
10 (n = 3)	B	D	A	C
11 (n = 3)	C	A	D	B
12 (n = 3)	D	C	B	A
**Treatment** **	**BIA 28–6156**	**Placebo**	**Moxifloxacin**	
A	1 × 60 mg	2 x	‐	
B	2 × 60 mg + 1 × 30 mg	‐	‐	
C	‐	3 x	‐	
D	‐	2 x	1 × 400 mg	

n = number of subjects.

A, B, C, and D  =  1 of 4 possible study drug treatments.

* One subject was withdrawn from the study and only received the placebo treatment in Period 1. This subject was replaced, and the replacement subject received all planned treatments according to the treatment sequence.

** All treatments administered in the fed state.

On each dosing day (Day 1 of each of the four treatment periods), subjects were administered a single dose of one of 60 mg BIA 28–6156 (1 × 60‐mg BIA 28–6156 capsule + 2 placebo capsules), 150 mg BIA 28–6156 (2 × 60‐mg BIA 28–6156 capsules + 1 × 30‐mg BIA 28–6156 capsule), 400 mg moxifloxacin (1 × 400 mg moxifloxacin encapsulated tablets + 2 placebo capsules), or placebo (3 placebo capsules) in the fed state, with a minimum of a 10‐day washout between dosing in each treatment period.

The oral capsules containing BIA 28–6156 or placebo and the encapsulated oral tablets containing moxifloxacin were indistinguishable in appearance and taste, and the number of capsules administered per treatment was the same.

The supratherapeutic dose of 150 mg BIA 28–6156 represented a 2.5‐fold increase compared with the potential higher therapeutic dose (60 mg BIA 28–6156). Moxifloxacin was selected as the positive control due to its well‐known prolongation of QT/QTc in humans.^27^, ^28^, ^29^

Thirty‐seven subjects were included in the enrolled and safety sets, whereas 36 subjects were included in the PK set (as one of the subjects only received a placebo and therefore was not included in the PK set). A sample size of 36 subjects was calculated to obtain 30 evaluable subjects from the PK set who completed the study. This would provide more than 96% power to exclude that BIA 28–6156 causes more than a 10 ms QTc effect at clinically relevant plasma levels.

Healthy subjects reported to the medical screening center/clinical sites for the eligibility screening within 3 weeks prior to (the first) drug administration.

Screening consisted of a discussion of informed consent, medical history, demographics, physical examination, vital signs, 12‐lead ECG, clinical laboratory tests (hematology, plasma biochemistry, urinalysis, viral serology, drugs of abuse screen, and pregnancy test), review of the eligibility criteria, and previous and concomitant medication.

Subjects were aged between 18 and 55 years and had a body mass index (BMI) of 18 to 32 kg/m^2^; women had not to be of childbearing potential (either because of surgery or because postmenopausal for at least 1 year) or, if of childbearing potential, use double‐barrier or intrauterine device pregnancy protection. No medication, other than the study medication, hormonal contraceptives (which could be used throughout the study), or SARS‐CoV‐2 vaccines are allowed up to 15 days before the first BIA 28–6156 dose. Concomitant medication or other therapy administered due to AEs was also allowed.

Subjects with abnormal baseline ECG, family history of arrhythmia, Torsades de Pointes, long QT syndrome, myocardial infarction, and atrioventricular block were excluded from study participation.

Written informed consent was obtained for each study participant.

The study drug was administered early in the morning at approximately 30 min after the start of breakfast, and subjects fasted for 4 h until lunch following the administration. Water drinking was allowed as desired except for 2 h before and 1 h after dosing. Twenty‐four hours before and after dosing, subjects were requested to abstain from alcohol and stimulating beverages containing xanthine derivatives (coffee or tea), citrus fruits or their juices, cranberries, and star fruit.

This study was designed and conducted according to the International Council for Harmonization (ICH) E14 guideline.^30^, ^31^, ^32^ The study was conducted according to the Helsinki Declaration, ICH Good Clinical Practice recommendations, and applicable local regulations. The study was approved by an independent ethics committee (Evaluation of Ethics in Biomedical Research, Assen, The Netherlands).

### Safety Assessments

Safety was analyzed through the incidence and severity of treatment emergent adverse events (TEAEs). Adverse events were coded according to the Medical Dictionary for Regulatory Activities (MedDRA, version 24.1). Any clinically significant observations in the results of clinical laboratory parameters, ECG, continuous cardiac monitoring, vital signs, or physical examination were recorded as AEs.

Any AE that occurred before starting the study was considered a pretreatment AE, unless it was associated with a study procedure, in which case it was to be reported as related to the study medication. In addition, all AEs reported spontaneously during the study were recorded.

### Blood Sampling and Plasma Drug Assays

Blood samples for pharmacokinetic (PK) analyses of BIA 28–6156 (4 mL) and its metabolites (2 mL) were drawn by direct venipuncture or intravenous catheter, during each period at pre‐dose and at 0.5, 1, 2, 3, 4, 5, 6, 8, 12‐, 24‐, 48‐, and 72‐h post‐dose.

### Bioanalysis of Analytes

Determination of BIA 28–6156 plasma concentrations was conducted in compliance with Good Laboratory Practice at the Bioanalytical Laboratory of ICON using validated liquid chromatography‐mass spectrometry/mass spectrometry methods.

The quantification of BIA 28–6156 and moxifloxacin was performed using a Triple Quad 5000 mass spectrometer equipped with a turbo ion spray source for detection in positive ion mode. Sample processing was performed through protein precipitation using a sample volume of 50.0 µL. Separation between the analyte and interfering endogenous compounds was achieved by UHPLC‐MS/MS using a Waters Acquity UPLC BEH C18 column (30 × 2.1 mm, 1.7 µm), operated at 60°C. For BIA 28–6156 a water:formic acid 98%–100% (100:0.1 v/v) solution as mobile phase A and acetonitrile as mobile phase B were used, operated at a gradient with an initial flow rate of 1.50 mL/min; for moxifloxacin, a Waters Acquity UPLC BEH C18 column (50 × 2.1 mm, 1.7 µm) at 60°C, using 0.1% formic acid in water as mobile phase A and acetonitrile as mobile phase B were used, operated at a gradient with an initial flow rate of 1.00 mL/min. Quantification was based on multiple reaction monitoring (MRM) of the transitions: m/z 359.2–174.1 for BIA 28–6156; m/z 365.3–180.1 for BIA 28‐d6; m/z 402.2–261.2 for moxifloxacin; m/z 406.2 – 263.2 for moxifloxacin‐d4. A linear calibration curve with a 1/x^2^ weighting factor was used, ranging from 10.0 to 20,000 ng/mL for BIA 28–6156, and ranging from 5.00 to 5000 ng/mL for moxifloxacin, in human plasma.

The results from calibration samples and quality control samples demonstrated acceptable performance of the methods throughout the experimental period. Data on the performance of the method and stability indicate that the sample results, as reported, are reliable.

### ECG Interpretation

The continuous cardiac monitoring data (Holter recordings) were analyzed by ERT/Clario, Philadelphia, US. ECG analysts were blinded to the subject, visit (time and day), and treatment allocation.

The primary analysis used concentration‐QTc modeling to investigate the relationship between plasma concentrations of BIA 28–6156 and the change in QTcF from baseline (ΔQTcF) to exclude the possibility of a placebo‐corrected ΔQTcF exceeding 10 ms at clinically relevant plasma levels, as shown by the upper bound of the two‐sided 90% confidence interval (CI) of the model‐predicted QTc effect (ΔΔQTcF) at the observed geometric mean C_max_ of BIA 28–6156 in the study. This power was estimated approximately using a paired t‐test. The calculation assumes a 1‐sided 5% significance level, a small underlying effect of BIA 28–6156 of 3 ms, and a standard deviation of the ΔQTcF of 8 ms for both BIA 28–6156 and placebo.

Additionally, the effects of BIA 28–6156 on placebo‐corrected ΔQTcF, ΔHR, ΔPR, and ΔQRS (ΔΔQTcF, ΔΔHR, ΔΔPR, and ΔΔQRS) were assessed at each post‐dosing time point.

Categorical outliers were identified for placebo‐corrected changes from baseline in QTcF, heart rate, PR interval, QRS interval, T‐wave morphology, and U‐wave presence. Assay sensitivity was assessed by performing a concentration‐QTc analysis of the effect of moxifloxacin on ΔΔQTcF, using a similar modeling approach to that of the primary analysis.

### Analyses

#### Pharmacokinetic Analysis

The PK parameters and endpoints included the maximum observed plasma concentration (C_max_), time at which C_max_ was observed (t_max_), area under the plasma concentration–time curve (AUC) from zero to the last quantifiable drug concentration (AUC_0–t_) and from zero to infinity (AUC_0–inf_), and the apparent terminal half‐life (t_1/2_).

#### Statistical Analysis

The Biostatistics Department of ICON generated a statistical analysis plan (SAP) for the PK and safety analysis, and a separate SAP was generated by ERT/Clario for the continuous cardiac monitoring evaluation.

Assay sensitivity was considered adequate if the slope of the concentration‐QTc relationship (ΔQTcF) was statistically significant at a 10% significance level (two‐sided test) and if the predicted QT effect (i.e., the lower bound of the two‐sided 90% CI of ΔΔQTcF) exceeded 5 ms at the observed geometric mean C_max_ of 400 mg moxifloxacin.

The concentration‐QTc (cQTc) analysis followed the modeling principles and best practices outlined in the scientific white paper on concentration‐QTc modeling by Garnett et al. (2018), which is widely accepted as a reference framework for such analyses in regulatory settings. This methodology involves the use of linear mixed‐effects models to characterize the relationship between drug plasma concentration and change‐from‐baseline QTcF (ΔQTcF), allowing for robust estimation of drug‐induced QT prolongation at relevant plasma concentrations.^33^ For additional model independent checks information, see Figures S1–S4, Supporting Information.

## Results

### Populations

A total of 37 subjects (17 females and 20 males) were enrolled. These included one replacement subject who received all planned treatments according to the treatment sequence (placebo). One subject was withdrawn from the study (found positive for benzodiazepine at the drug screen on Day‐1 of Period 2). Therefore, all 37 subjects were included in the enrolled and safety sets, whereas 36 subjects were included in the PK set. Thirty‐three subjects completed the study. The mean age was 37 years and the mean BMI was 25.1 kg/m^2^.

### Pharmacokinetics

Median plasma BIA 28–6156 t_max_ and geometric mean plasma BIA 28–6156 t_½_ were similar between the 60 and 150 mg doses of BIA 28–6156: median plasma (range) BIA 28–6156 t_max_ was 3.01 h (0.50 – 5.02) and 4.00 h (1.00–8.02) and geometric mean plasma t_1/2_ was 29.6 h (12.7–57.2) and 30.5 h (10.5–64.3), respectively. Exposure was higher following 150 mg BIA 28–6156 compared to 60 mg BIA 28–6156, with geometric mean values of 3598 ng/mL (2000–7150) and 1617 ng/mL (958–3430) for C_max_, and 101,853 ng h/mL (56,352–194,899) and 41,005 ng h/mL (22,081–72,948) for AUC_0–t_, respectively (Table 2). Median plasma moxifloxacin t_max_ was 3.0 h (0.50–5.02) and geometric mean moxifloxacin t_½_ was 13.1 h (9.11–16.5) (Figure S5, Supporting Information).

**Table 2:** Geometric Mean (%CV) of BIA 28–6156 and Moxifloxacin Plasma Pharmacokinetic Parameters (PK Set).

Parameter	60 mg BIA 28–6156 (N = 34)	150 mg BIA 28–6156 (N = 36)	400 mg Moxifloxacin (N = 33)
C_max_ (ng/mL)	1617 (28.6)	3598 (27.2)	1899 (21.0)
t_max_ (h)^a^	3.01 (0.50–5.02)	4.00 (1.00–8.02)	3.00 (0.50–5.02)
AUC_0‐last_ (ng.h/mL)	41,005 (30.0)	101,853 (27.6)	27,508 (20.0)
AUC_0‐inf_ (ng.h/mL)	52,073 (39.1)	131,488 (36.8)	28,212 (20.7)
t_½_ (h)^b^	31.8 (11.8)	33.0 (13.0)	13.2 (1.82)

AUC_0‐last_, area under the plasma concentration versus time curve (AUC) from time zero to the time of the last measurable concentration; AUC_0‐inf_, AUC from time zero extrapolated to infinity; C_max_, maximum observed plasma drug concentration; CV, coefficient of variation; t_max_, time of maximum observed plasma drug concentration; t_½_, terminal elimination half‐life.

^a^For t_max_ the median (range) is presented instead of geometric mean (%CV).

^b^For t_½_ the arithmetic mean (standard deviation) is presented instead of geometric mean (%CV).

### Continuous Cardiac Monitoring and Safety Results

The highest mean plasma concentration of BIA 28–6156 was achieved 4 h after participants were administered either the 60 or 150 mg doses. Following the administration of BIA 28–6156 at both dose levels, ΔHR mirrored the pattern observed with placebo, resulting in minimal ΔΔHR. The maximal observed mean ΔΔHR was 2.4 bpm, occurring 4 h after administration of the 150 mg dose. BIA 28–6156 did not show a clinically significant impact on cardiac conduction, as shown by stable PR and QRS intervals.

**Figure 1:**
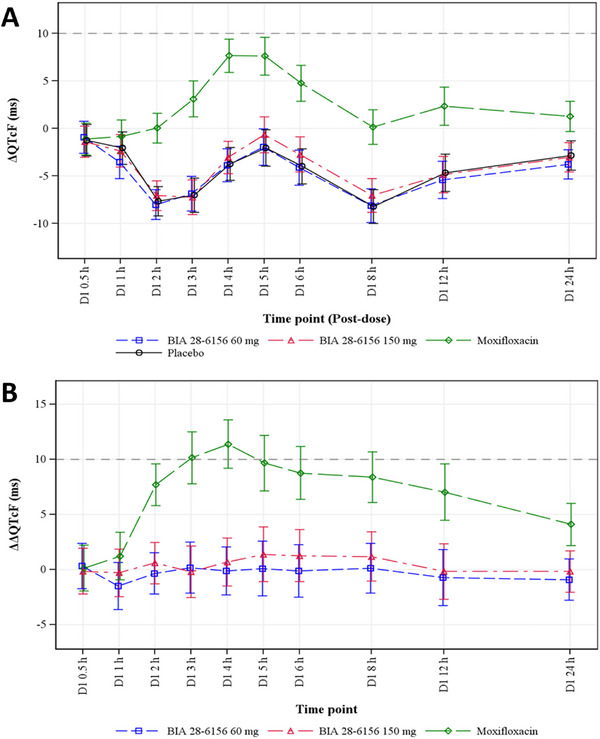
(A) Change‐from‐baseline QTcF (ΔQTcF) across time point (QT/QTc analysis set). (B) Placebo‐corrected change‐from‐baseline QTcF (ΔΔQTcF) across time point (QT/QTc analysis set). LS mean and 90% CI based on a linear mixed‐effects model: ΔQTcF = Time + Treatment + Time × Treatment + Baseline QTcF + Period + Sequence. An unstructured covariance structure was used to specify the repeated measures (post‐dose time points for subject within treatment period). The model also includes a subject‐specific random effect.

ΔQTcF for both doses of BIA 28–6156 closely paralleled the placebo response, leading to narrow ΔΔQTcF fluctuations, within ±1.5 ms across all post‐dose time points (Figure 1A). In contrast, administration of moxifloxacin resulted in QTc prolongation, with a maximum mean ΔΔQTcF of 11.4 ms observed 4 h post‐dose (Figure 1B).

A concentration‐QTc analysis using a linear model with a treatment effect‐specific intercept was performed for BIA 28–6156 plasma concentrations. The slope of the BIA 28–6156 concentration‐QTc relationship was shallow and not statistically significant (0.00015 ms per ng/mL; 90% CI: −0.000309 to 0.000600). Additionally, the treatment effect‐specific intercept was small and not statistically significant (−0.11 ms). (Figures S6 and S7, Supporting Information).

Predicted effects on ΔΔQTcF at the geometric mean peak plasma concentrations of BIA 28–6156 were 0.13 ms (90% CI: −0.61 to 0.87) for the 60 mg dose (1616.8 ng/mL) and 0.41 ms (90% CI: −1.00 to 1.82) for the 150 mg dose (3532.6 ng/mL). Based on this analysis, a QTcF effect (ΔΔQTcF) exceeding 10 ms was excluded across the entire observed range of BIA 28–6156 plasma concentrations, up to approximately 7150 ng/mL.

The same linear model with a treatment effect‐specific intercept was employed in the assay sensitivity analysis for moxifloxacin. In this case, the slope of the concentration‐QTc relationship was positive and statistically significant (0.0044 ms per ng/mL; 90% CI: 0.00316 to 0.00558), and the lower bound of the 90% CI for the predicted QT effect at the geometric mean peak moxifloxacin concentration (1894.0 ng/mL) was 10.20 ms (90% CI: 8.33 to 12.07), confirming assay sensitivity.

### Tolerability

A total of 90 TEAEs were reported in 24 of 37 (64.9%) subjects during the study, and 88 of these TEAEs were transient and had resolved without sequelae by the follow‐up visit. The System Organ Class (SOC) “General Disorders and Administration Site Conditions” had the greatest number of TEAEs, followed by “Nervous System Disorders”, “Musculoskeletal and Connective Tissue Disorders”, and “Respiratory, Thoracic, and Mediastinal Disorders” (Table 3).

**Table 3:** Summary of Treatment Emergent Adverse Events by SOC

System organ class	60 mg BIA 28–6156 (N = 34) E/n (%)	150 mg BIA 28–6156 (N = 36) E/n (%)	400 mg Moxifloxacin (N = 34) E/n (%)	Placebo (N = 35) E/n (%)	Overall (N = 37) E/n (%)
Any adverse events	19/9 (26.5)	27/14 (38.9)	25/10 (29.4)	19/14 (40.0)	90/24 (64.9)
General disorders and administration site conditions	7/4 (11.8)	8/5 (13.9)	13/6 (17.6)	14/13 (37.1)	42/16 (43.2)
Nervous system disorders	3/3 (8.8)	5/5 (13.9)	3/3 (8.8)	2/2 (5.7)	13/8 (21.6)
Musculoskeletal and connective tissue disorders	2/2 (5.9)	1/1 (2.8)	3/2 (5.9)	0	6/5 (13.5)
Respiratory, thoracic, and mediastinal disorders	3/2 (5.9)	3/2 (5.6)	0	0	6/4 (10.8)

%, number of subjects (n) as a percentage of number of subjects (N) per treatment; E = number of adverse events; SOC = system organ class; N, number of subjects exposed; n, number of subjects that experienced the adverse events.

The most frequently reported TEAEs by preferred term (PT) (>10%) were catheter site‐related reaction, headache, fatigue, and catheter site pain (Table 4).

**Table 4:** Summary of treatment emergent adverse events by preferred term (>10%)

Preferred Term	60 mg BIA 28–6156 (N = 34) E/n (%)	150 mg BIA 28–6156 (N = 36) E/n (%)		400 mg Moxifloxacin (N = 34) E/n (%)	Placebo (N = 35) E/n (%)	Overall (N = 37) E/n (%)
Catheter site related reaction	4/3 (8.8)	2/1 (2.8)		2/2 (5.9)	5/4 (11.4)	13/8 (21.6)
Headache	3/3 (8.8)	5/5 (13.9)		3/3 (8.8)	2/2 (5.7)	13/8 (21.6)
Fatigue	1/1 (2.9)	0		2/2 (5.9)	2/2 (5.7)	5/4 (10.8)
Catheter site pain	0	1/1 (2.8)		2/2 (5.9)	1/1 (2.9)	4/4 (10.8)

%, number of subjects (n) as a percentage of number of subjects (N) per treatment; E, number of AEs; MedDRA, Medical Dictionary for Regulatory Activities; SOC, system organ class; N, number of subjects exposed; n = number of subjects that experienced the AE.

Most TEAEs were mild in severity; 1 TEAE was of Grade 2 (moderate) severity (dental pulpitis considered unrelated to the study drug), and 1 TEAE was of Grade 3 (severe) severity (hypersensitivity considered related to moxifloxacin). Two subjects were withdrawn from the study because of TEAEs: one subject was withdrawn because of increased ALT and GGT after receiving the 150 mg dose of BIA 28–6156, which were considered of Grade 1 (mild) severity and related to BIA 28–6156; and one subject was withdrawn because of hypersensitivity (Grade 3 in severity), pollakiuria (Grade 1 in severity), and nervousness (Grade 1 in severity) after receiving 400 mg moxifloxacin.

Two TEAEs (cough and injection site muscle weakness) were still ongoing at follow‐up. They were both mild in severity and considered unrelated to BIA 28–6156 or moxifloxacin.

Of 90 TEAEs, nine TEAEs reported by five (13.5%) subjects were considered related to the study drug. Of these nine TEAEs, four were reported by two (5.6%) subjects receiving 150 mg BIA 28–6156 (headache and nausea reported by one subject, and increased alanine aminotransferase (ALT) and increased gamma glutamyl transferase (GGT) reported by another subject).

No deaths or other SAEs were reported.

There were no clinically significant safety observations in vital sign measurements, physical examinations, safety ECG parameters (Table S1, Supporting Information), or laboratory values that were reported during the study and considered related to BIA 28–6156 other than increased ALT and increased GGT in one subject after 150 mg BIA 28–6156.

## Discussion

This study conducted a comprehensive QT/QTc evaluation to determine whether BIA 28–6156 affects cardiac repolarization following ICH E14 guidelines. As per these guidelines, this study was intended to be conducted early in clinical development, to provide maximum guidance for later clinical trials and to determine whether the effect of BIA 28–6156 on the QT/QTc interval in PD patients should be studied intensively during later stages of drug development. Also, according to ICH E14, moxifloxacin was selected as the positive control due to its well‐known prolongation of QT/QTc in humans. Its consistent QT effect, combined with a favourable cardiovascular safety profile, has made moxifloxacin the positive control of choice for thorough QT/QTc studies.^28^, ^30^, ^31^, ^34^

The primary analysis focused on concentration‐QTc modeling of the relationship between BIA 28–6156 plasma concentrations and ΔQTcF, aiming to rule out an effect of ΔΔQTcF >10 ms at clinically relevant plasma levels.

Of note, Fredericia's correction formula (QTcF) was used in this study as it was deemed the most appropriate method for correcting measurements of QT intervals considering heart rate variability. QTcF is considered the most accurate correction across a broad range of heart rates compared to other commonly used methods, such as Bazett's formula (QTcB), which tends to overcorrect at higher heart rates and undercorrect at lower ones.

The crossover design of the study was particularly beneficial in reducing the impact of inter‐subject variability on the observed treatment effect, minimizing the number of subjects required for adequate statistical power.

Plasma concentrations higher than those following the intake of therapeutic doses should be tested in a thorough QT/QTc study.

In this study, a supratherapeutic dose of 150 mg of BIA 28–6156 was tested, which is 2.5 times higher than the potential higher therapeutic dose of 60 mg. The no‐observed‐adverse‐effect levels (NOAELs) from toxicology studies were 60 mg/kg in female rats and 120 mg/kg in male and female dogs. The human equivalent dose for the rat NOAEL was 9.7 mg/kg, which translates to 582 mg for a 60‐kg human, offering a ≈4‐fold safety margin compared to the 150 mg supratherapeutic dose. The rat NOAEL of 60 mg/kg resulted in a C_max_ of 39.5 µg/mL, while the highest human dose of 90 mg produced a C_max_ of ≈3 µg/mL. Since dose proportionality was observed, the 150 mg dose in humans would likely result in a C_max_ below 5 µg/mL, which is still about eight times lower than the rat NOAEL C_max_. The use of a supratherapeutic dose within acceptable safety margins allowed to cover for the worst‐case scenarios that potentially lead to higher drug levels in the body and an increased risk of side effects like QT/QTc prolongation in potential situations that may lead to high clinical exposure for BIA 28–6156. These include accumulation of the analytes that may be caused by intrinsic factors, such as age (metabolism and organ function can change as people age, potentially leading to higher drug levels in the body); special populations (pregnant women, children, elderly individuals, people with pre‐existing health conditions, in whom drugs might be metabolized differently); or by extrinsic factors, such as potential drug–drug interactions or repeated dosing.

Results indicate that BIA 28–6156, at both the therapeutic and supratherapeutic doses, did not produce any clinically relevant effects on the QTc interval or other ECG parameters. The concentration‐QTc analysis effectively excluded the possibility of a ΔΔQTcF effect exceeding 10 ms within the full observed range of BIA 28–6156 plasma concentrations, which reached up to approximately 7150 ng/mL. Moxifloxacin, as expected, demonstrated a statistically significant increase in ΔΔQTcF exceeding 5 ms, thereby confirming the assay's sensitivity to detect changes in cardiac conduction parameters.

The administration of BIA 28–6156 at both 60 and 150 mg doses, along with placebo and 400 mg moxifloxacin, was generally well‐tolerated in healthy male and female subjects. There were no clinically significant findings in terms of vital signs, ECG results, or physical examination.

In conclusion, this study provides evidence that BIA 28–6156, at both the therapeutic and supratherapeutic doses, has no clinically relevant effects on cardiac parameters, constituting a negative QT/QTc study. Since PD patients are often elderly and may have comorbidities or polypharmacy concerns, the observed cardiac safety profile is particularly important. Ensuring cardiac safety without unnecessarily halting drug development is key to bringing effective PD treatments to market.

## Conflicts of Interest

This study was sponsored by BIAL R&D Investments, S.A. All authors were involved in the design or conduct of the study, the collection, management, or analysis of the data, and preparation of the article. Isa Peixoto, Teresa Carvalho, João Reis, Helena Gama, and Joerg Holenz are employees of BIAL‐Portela & C^a^, S.A. and report no additional conflicts of interest. Dana Hilt reports consulting fees from Recognify Therapeutics, Brenig Therapeutics, Actinogen Medical, and Kynexis Therapeutics. She also reports participation on Data Safety Monitoring Boards or advisory boards for Recognify Therapeutics, Brenig Therapeutics, Progentos Therapeutics, and Kynexis Therapeutics. She is a former employee of the BIAL group (BIAL‐Portela & C^a^, S.A.).

## Funding

The study was funded and conducted by BIAL – Investments.

## Supporting information



Supporting Information

## Data Availability

The data that support the findings of this study are available in the supplementary material of this article.
