# Supplementary material for: A Randomized, Double‐Blinded, Placebo‐Controlled QTc Study to Evaluate BIA 28–6156 Effect on Cardiac Repolarization in Healthy Volunteers
Source: Clin Pharmacol Drug Dev. 2026 Apr 9;15:e70055. doi: 10.1002/cpdd.70055 (PMC13065933; doi:10.1002/cpdd.70055)

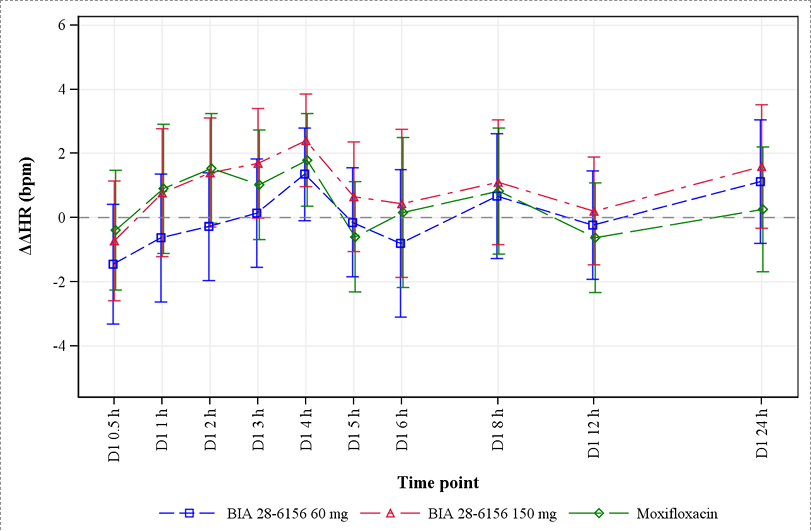


**Figure S1.** **Placebo-corrected change-from-baseline HR across time point (QTQTc analysis set).** LS mean and 90% CI based on a linear mixed-effects model: ΔHR = Time + Treatment + Time×Treatment + Baseline HR + Period + Sequence. An unstructured covariance structure was used to specify the repeated measures (post-dose time points for subject within treatment period). The model also includes a subject-specific random effect.


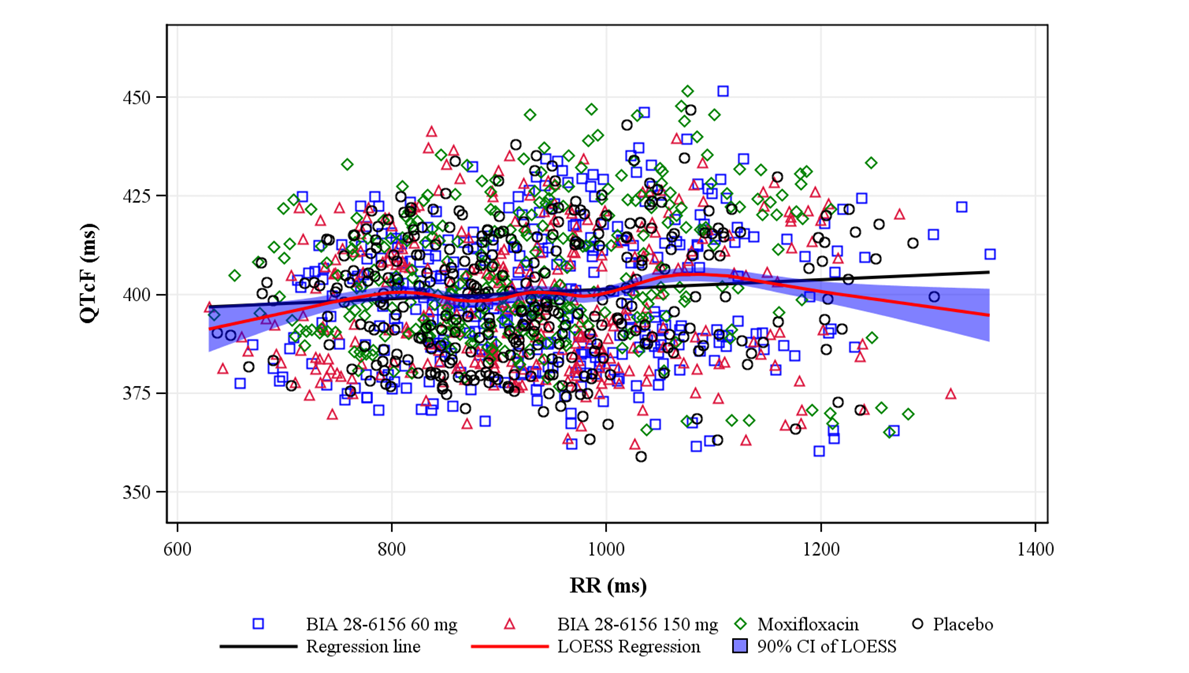


**Figure S2. a)** **Scatter plot of QTcF versus RR by treatment (QT/QTc analysis set).** The solid black line denotes the simple linear regression across all pairs of (RR,QTc). This line is based on the equation: QTcF = 389.28 + 0.012 ×RR with a P value of 0.0008 for the slope.

**
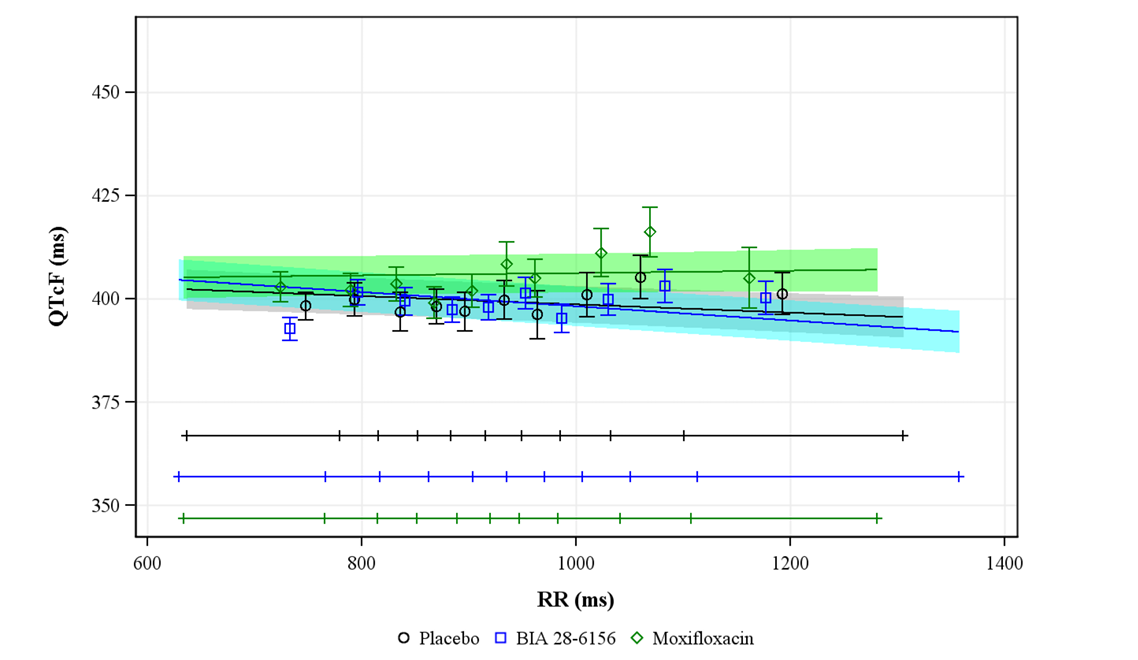
Figure S2. b) QTcF-RR decile plot by treatment (QT/QTc analysis set).** The grey, blue, and green shaded areas denote the 90% CI from the linear mixed effect model QTcF ≈ intercept + slope ×RR. The black circles, blue squares, and green diamonds with vertical bars denote the observed mean QTcF with 90% CI displayed at the median RR within each decile for placebo, BIA 28-6156, and Moxifloxacin, respectively. The black, blue, and green solid lines below the shaded area represent the deciles of observed RR values. The slope for placebo was -0.010 with a P value of 0.0059. The slope for BIA 28-6156 was -0.017 with a P value of < 0.0001. The slope for Moxifloxacin was 0.0028 with a P value of 0.4908.


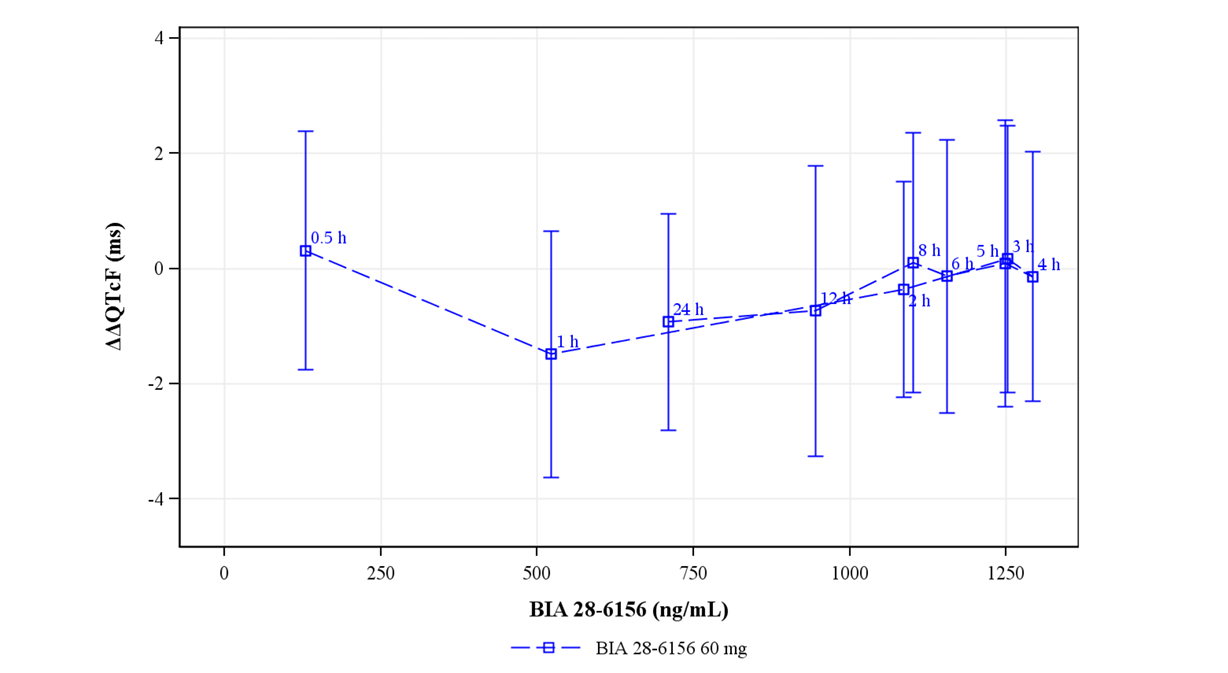


**Figure S3. a) Hysteresis plot of BIA 28-6156 plasma concentration and ΔΔQTcF connected in temporal order by dose (60 mg) (QT/QTc analysis set, PK/QTc analysis set).** ΔΔQTcF with 90% CI is from by-time point statistical modeling while concentration shows the mean from descriptive statistics. The labeled points on the figure denote the time points at which the (x,y) pairs occurred. The baseline time point (0,0) was included to not lose the period between dosing and the first measurement.


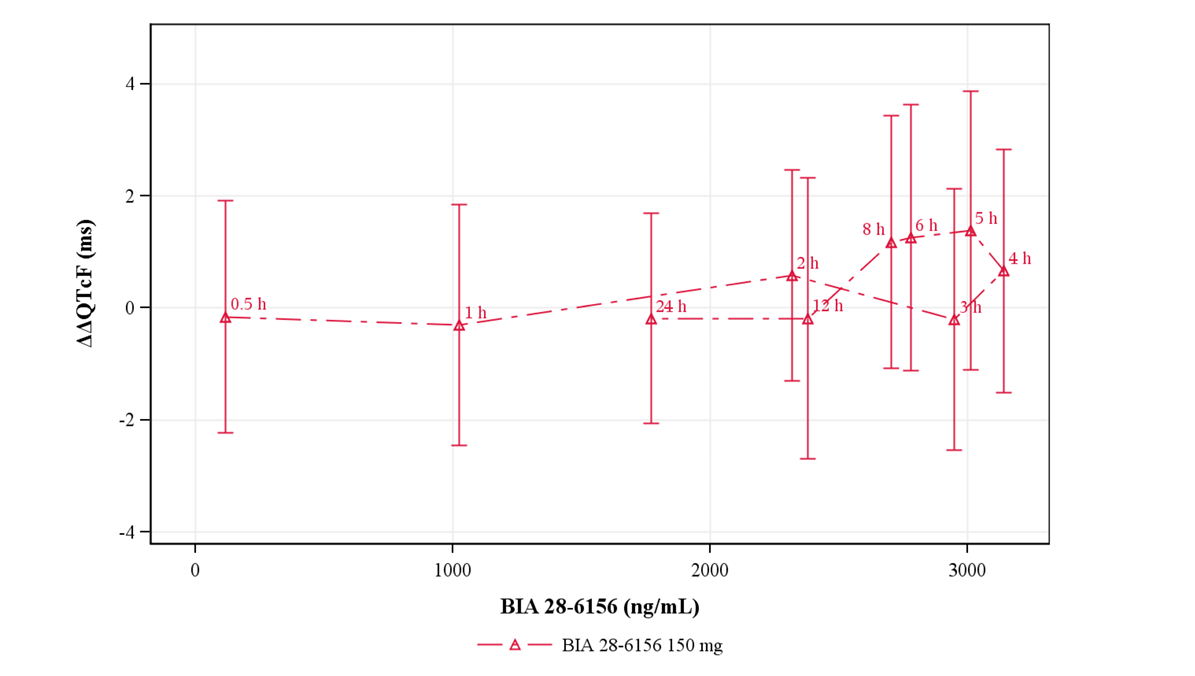
**Figure S3. b) Hysteresis plot of BIA 28-6156 plasma concentration and ΔΔQTcF connected in temporal order by dose (150 mg) (QT/QTc analysis set, PK/QTc analysis set).** ΔΔQTcF with 90% CI is from by-time point statistical modeling, while concentration shows the mean from descriptive statistics. The labeled points on the figure denote the time points at which the (x,y) pairs occurred. The baseline time point (0,0) was included to not losing the period between dosing and the first measurement.


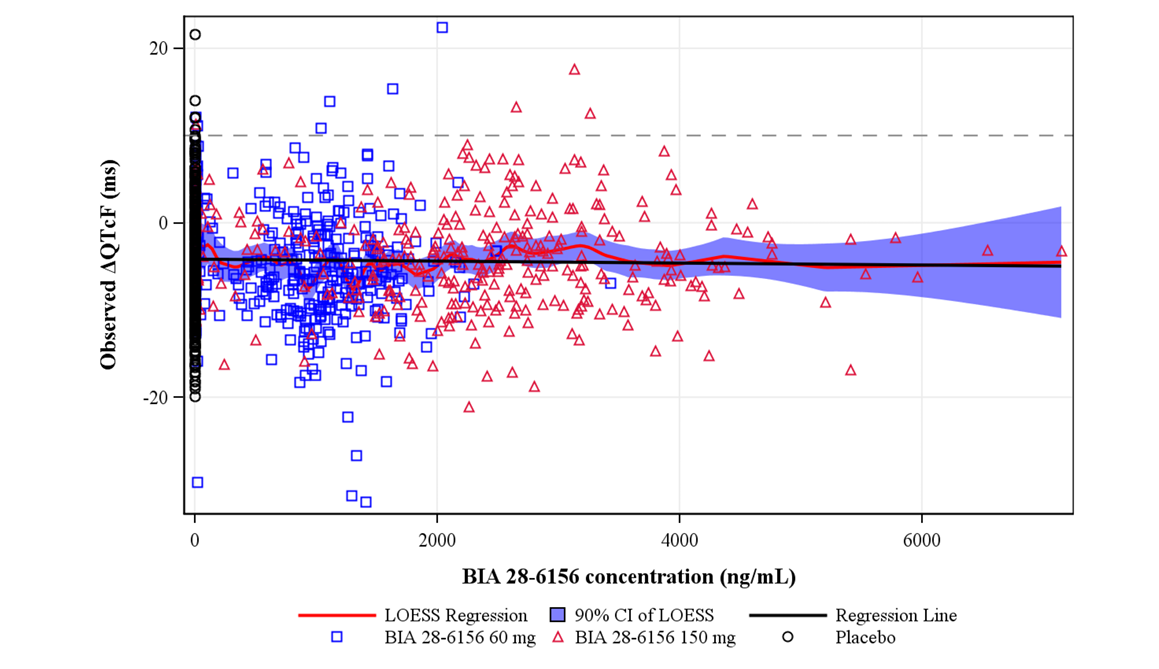


**Figure S4. Scatter plot of observed BIA 28-6156 plasma concentrations and ΔQTcF with simple linear regression and LOESS regression (PKQTc analysis set).** The red line with the blue shaded area denotes the LOESS regression and 90% confidence limits. The black solid line denotes the simple linear regression line. The plotted points denote the pairs of observed BIA 28-6156 plasma concentrations and ΔQTcF.


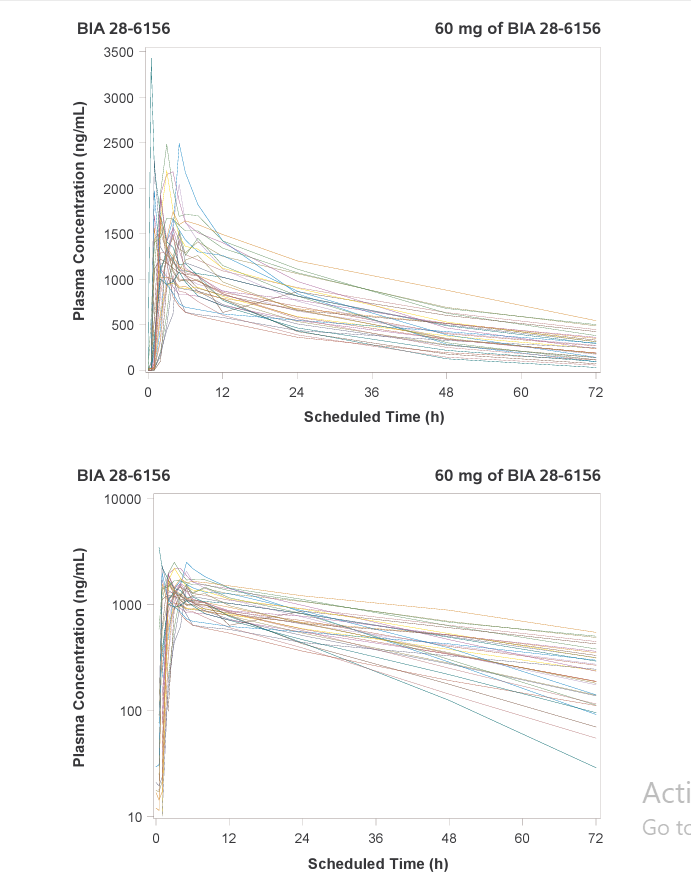


**Figure S5: Combined Individual BIA 28-6156 and Moxifloxacin Plasma Concentrations versus Time (Linear and Semi-Logarithmic Scale)**

**
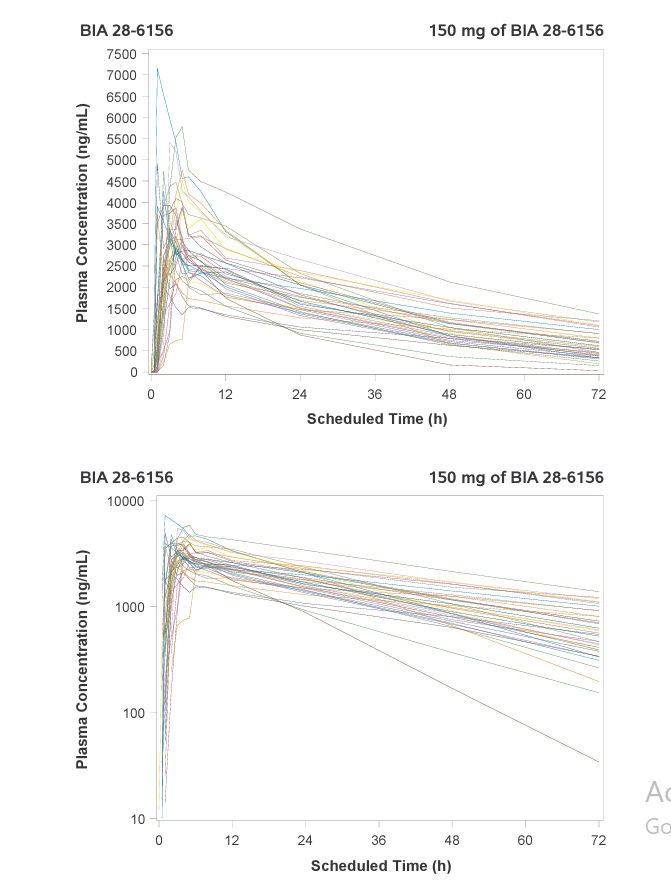
**

**Figure S5 (cont.): Combined Individual BIA 28-6156 and Moxifloxacin Plasma Concentrations versus Time (Linear and Semi-Logarithmic Scale) (cont.)**

**
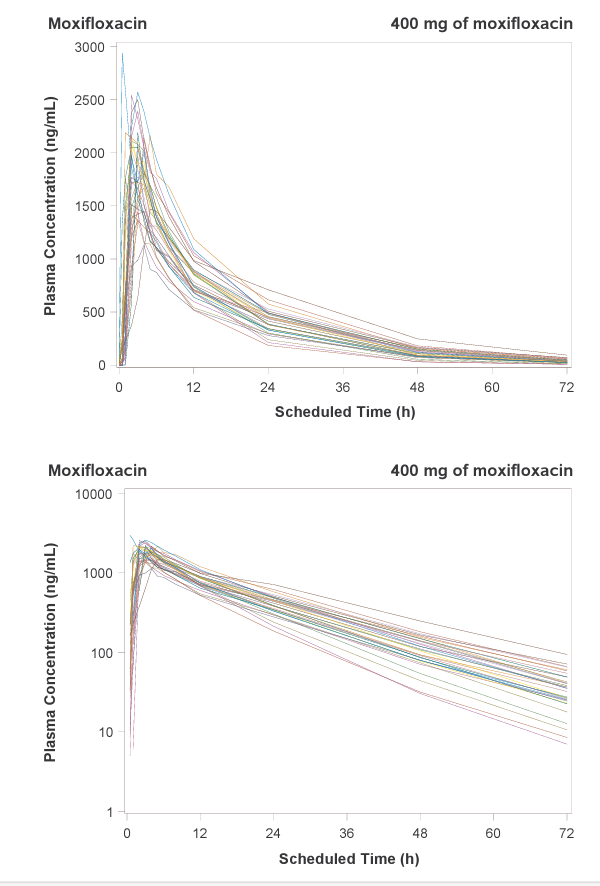
**

**Figure S5 (cont.): Combined Individual BIA 28-6156 and Moxifloxacin Plasma Concentrations versus Time (Linear and Semi-Logarithmic Scale)**

**
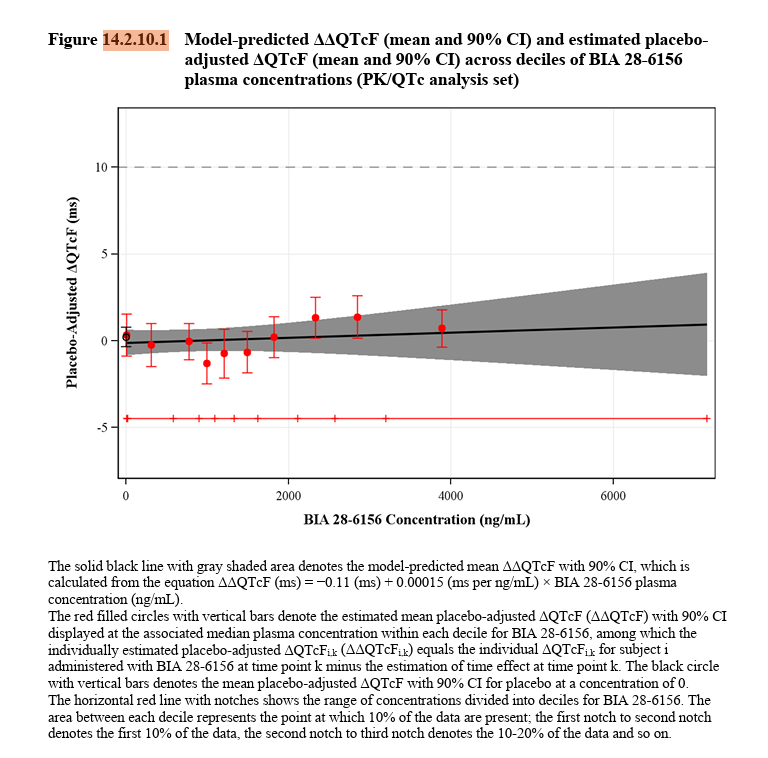
Figure S6: Model-predicted ΔΔQTcF (mean and 90% CI) and estimated placebo-adjusted ΔQTcF (mean and 90% CI) across deciles of BIA 28-6156 plasma concentrations (PK/QTc analysis set).** The solid black line with grey shaded area denotes the model-predicted mean ΔΔQTcF with 90% CI, which is calculated from the equation ΔΔQTcF (ms) = −0.11 (ms) + 0.00015 (ms per ng/mL) × BIA 28-6156 plasma concentration (ng/mL). The red-filled circles with vertical bars denote the estimated mean placebo-adjusted ΔQTcF (ΔΔQTcF) with 90% CI displayed at the associated median plasma concentration within each decile for BIA 28-6156, among which the individually estimated placebo-adjusted ΔQTcFi,k (ΔΔQTcFi,k) equals the individual ΔQTcFi,k for subject I administered with BIA 28-6156 at time point k minus the estimation of time effect at time point k. The black circle with vertical bars denotes the mean placebo-adjusted ΔQTcF with 90% CI for placebo at a concentration of 0. The horizontal red line with notches shows the range of concentrations divided into deciles for BIA 28-6156. The area between each decile represents the point at which 10% of the data are present; the first notch to second notch denotes the first 10% of the data, the second notch to third notch denotes the 10-20% of the data and so on.

**
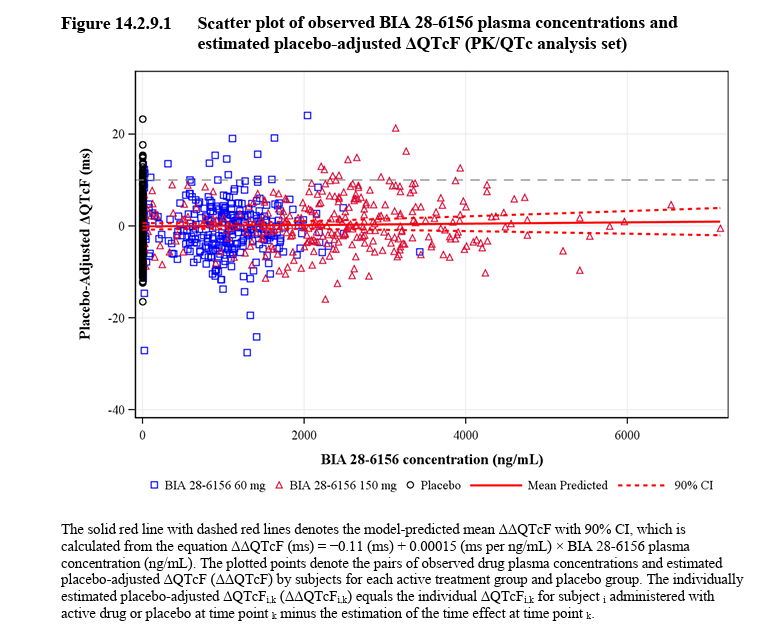
**

**Figure S7. Scatter plot of observed BIA 28-6156 plasma concentrations and**

**estimated placebo-adjusted ΔQTcF (PK/QTc analysis set).** The solid red line with dashed red lines denotes the model-predicted mean ΔΔQTcF with 90% CI, which is calculated from the equation ΔΔQTcF (ms) = −0.11 (ms) + 0.00015 (ms per ng/mL) × BIA 28-6156 plasma concentration (ng/mL). The plotted points denote the pairs of observed drug plasma concentrations and estimated placebo-adjusted ΔQTcF (ΔΔQTcF) by subjects for each active treatment group and placebo group. The individually estimated placebo-adjusted ΔQTcFi,k (ΔΔQTcFi,k) equals the individual ΔQTcFi,k for subject i administered with active drug or placebo at time point k minus the estimation of the time effect at time point k.

**
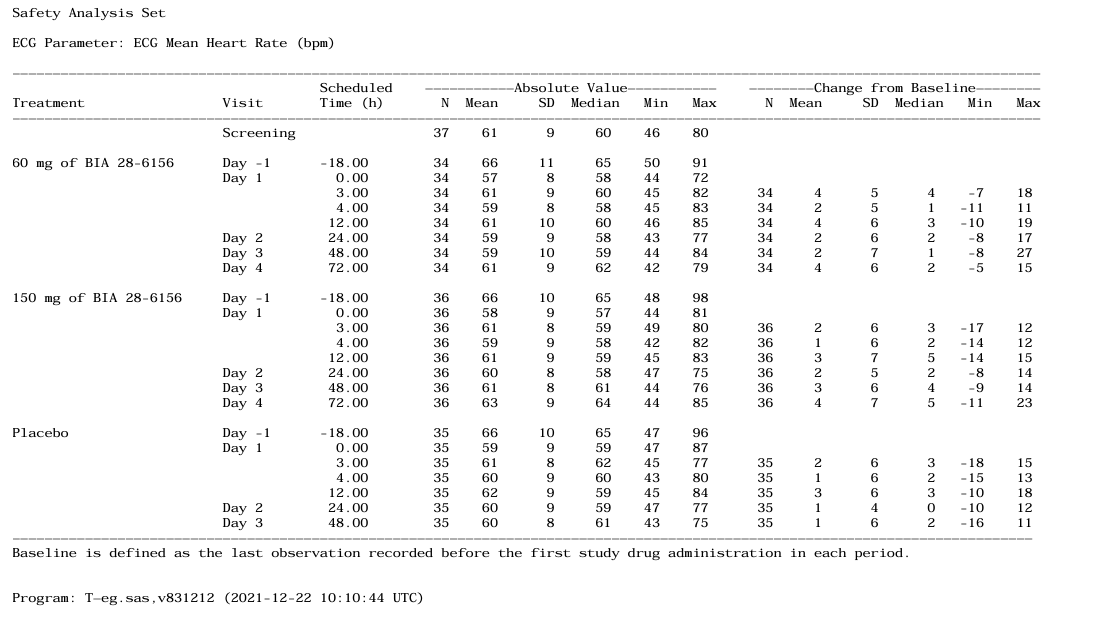
Table S1: Summary of 12-Lead Electrocardiogram**

**Table S1: Summary of 12-Lead Electrocardiogram
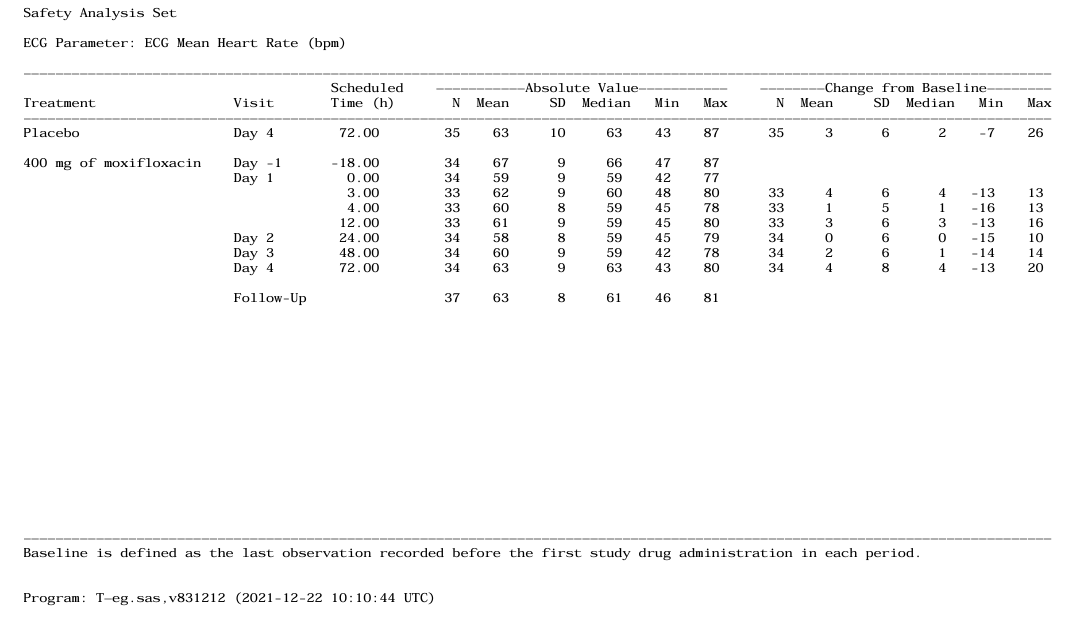
 (cont.)**

**Table S1: Summary of 12-Lead Electrocardiogram (cont.)**

**
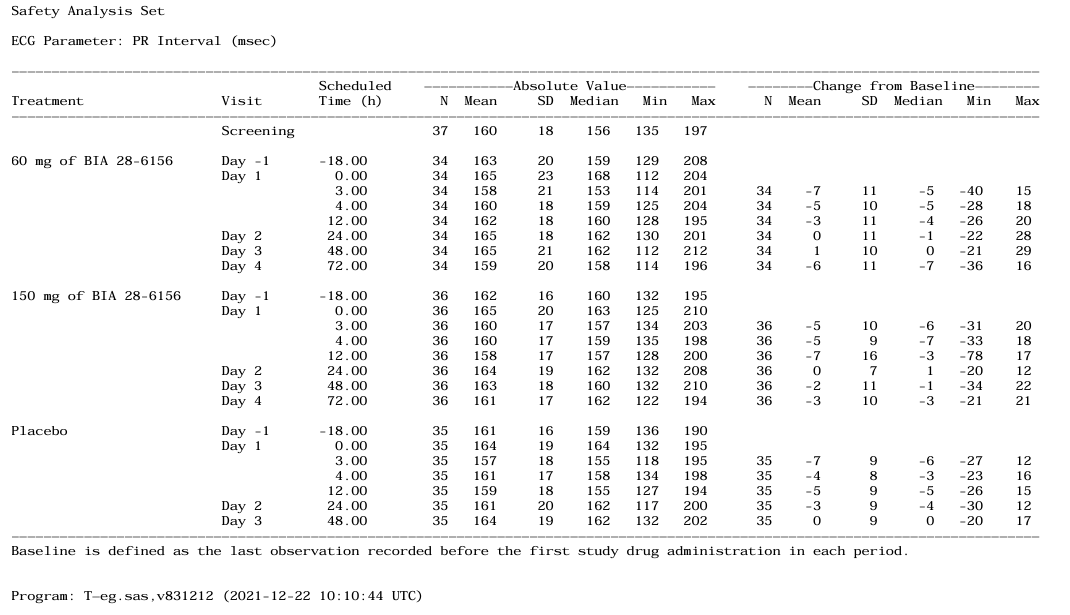
**

**Table S1: Summary of 12-Lead Electrocardiogram (cont.)**

**
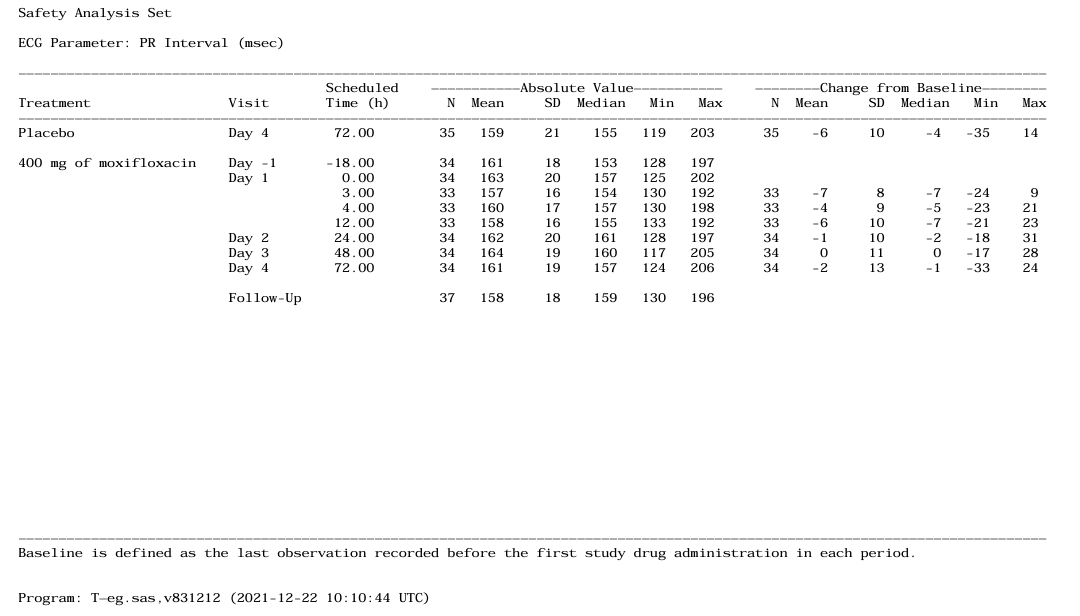
**

**Table S1: Summary of 12-Lead Electrocardiogram (cont.)**

**
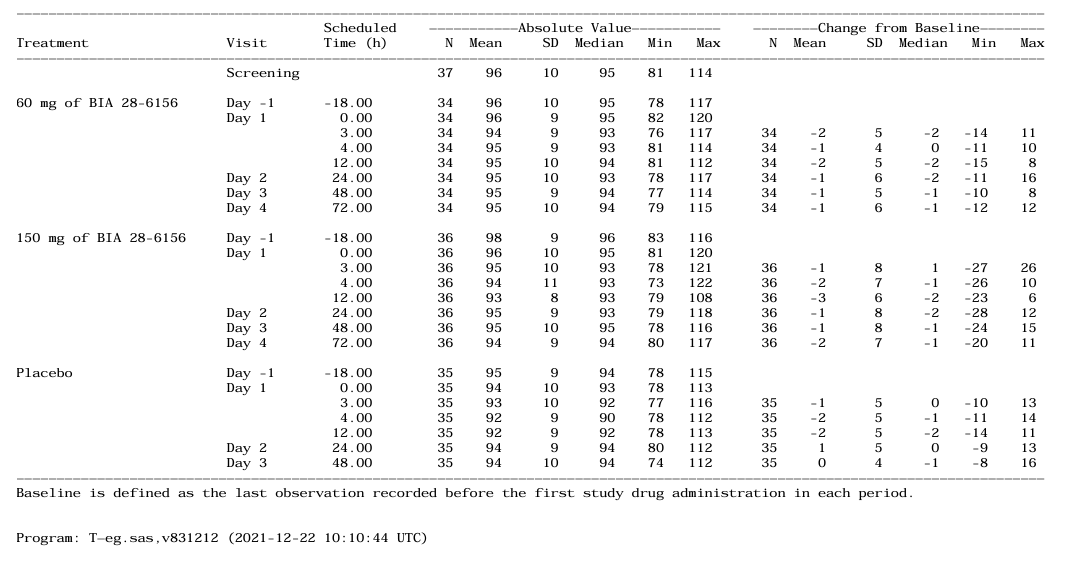
**

**Table S1: Summary of 12-Lead Electrocardiogram (cont.)**

**
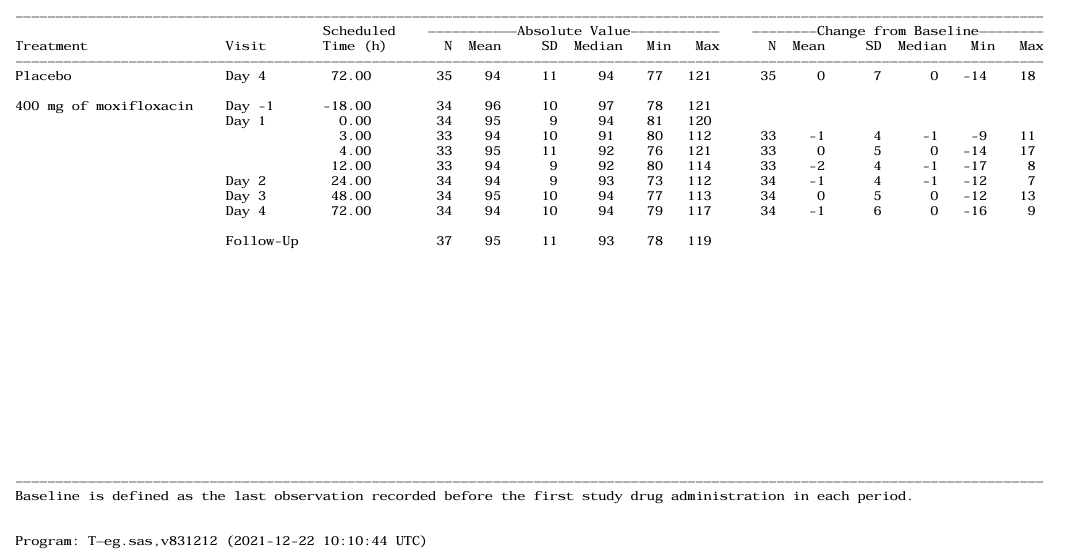
**

**
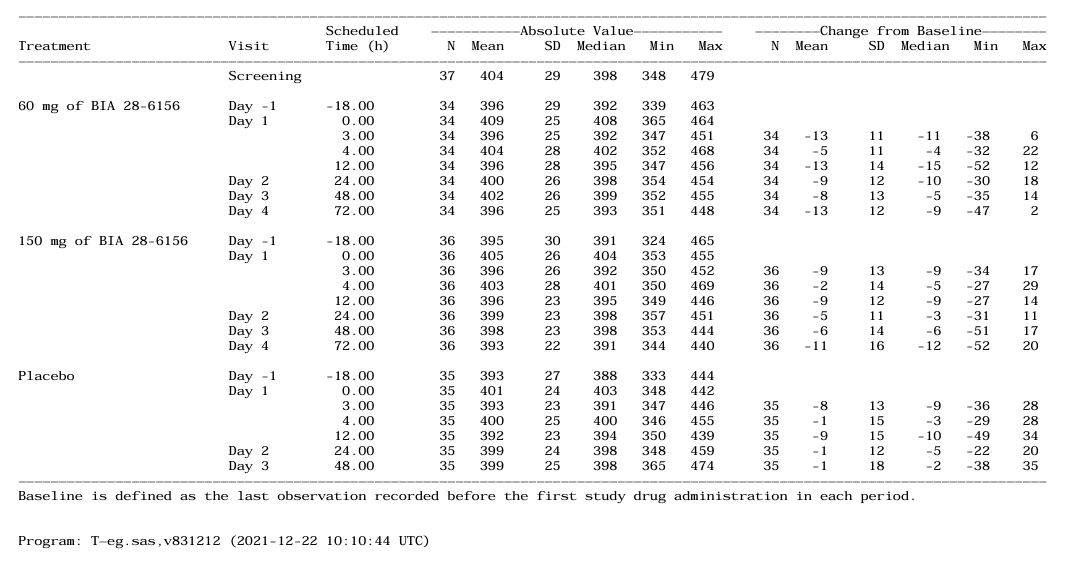
Table S1: Summary of 12-Lead Electrocardiogram (cont.)**

**
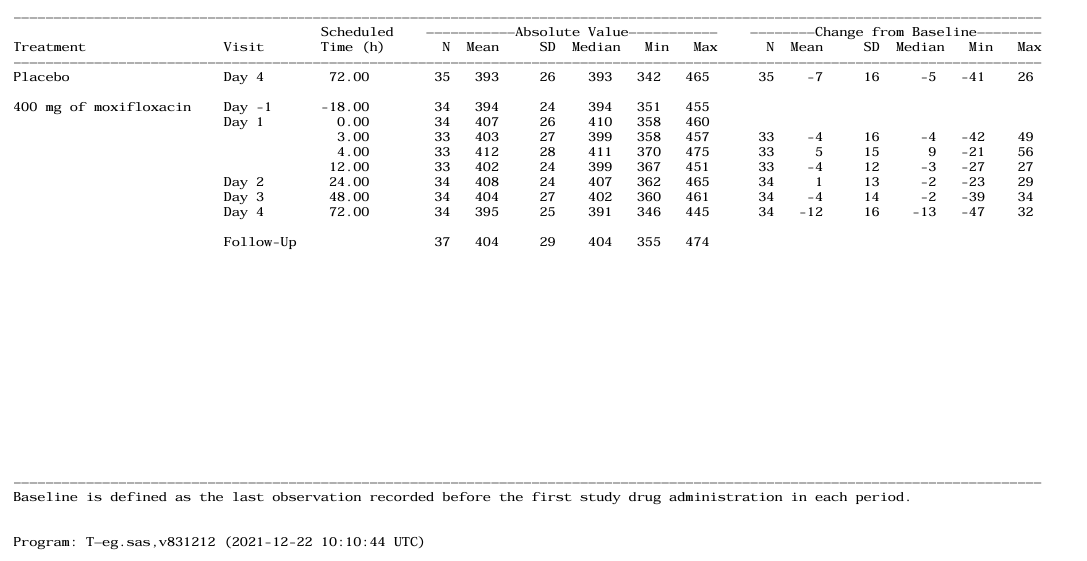
Table S1: Summary of 12-Lead Electrocardiogram (cont.)**

**Table S1: Summary of 12-Lead Electrocardiogram (cont.)**

**
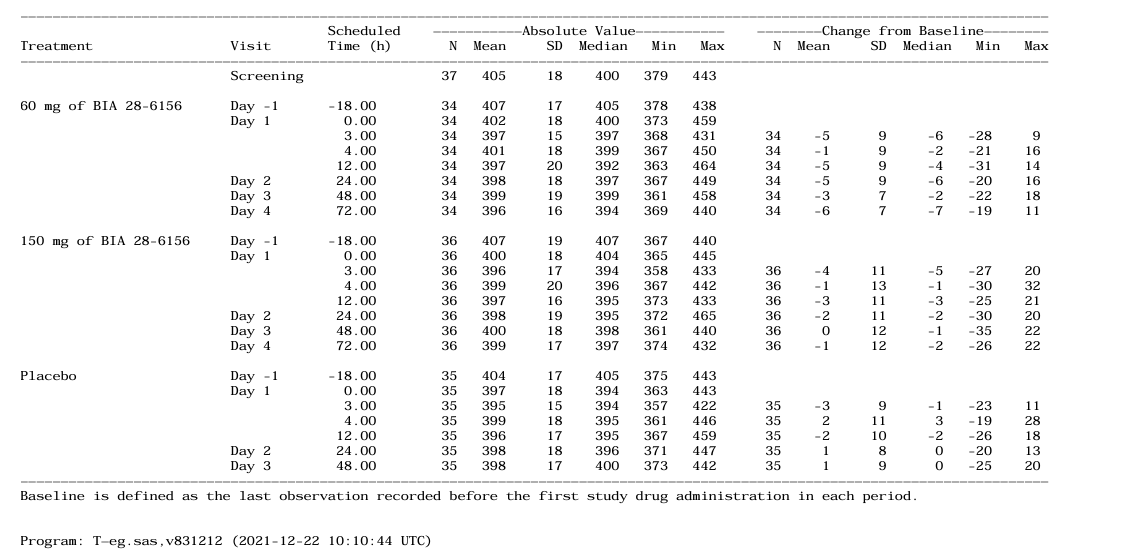
**

**Table S1: Summary of 12-Lead Electrocardiogram (cont.)**


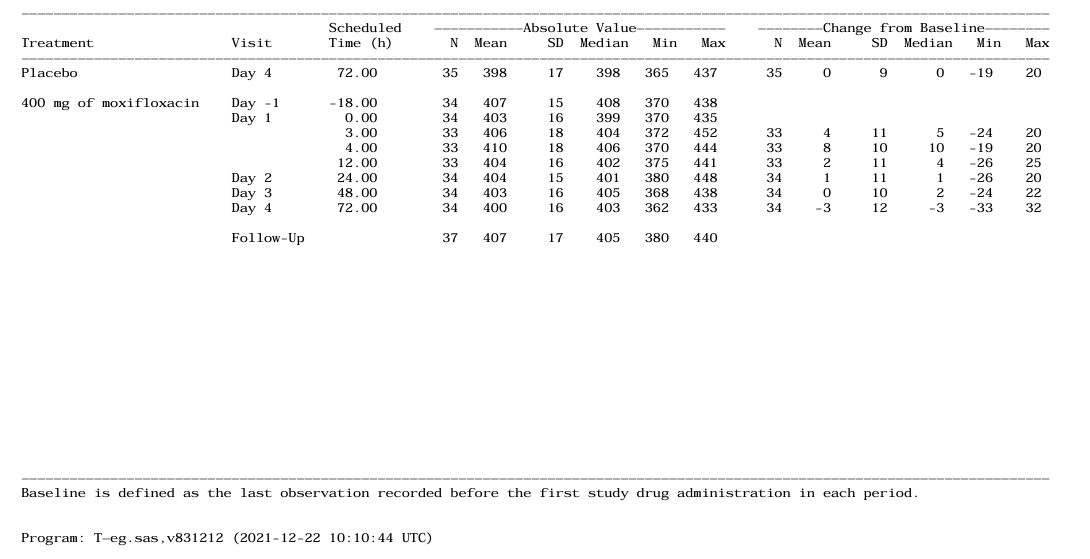

Supplement: Supplementary file 1 — Supporting Information [file CPDD-15-0-s001.docx]
